# Update on Antiviral Strategies Against COVID-19: Unmet Needs and Prospects

**DOI:** 10.3389/fimmu.2020.616595

**Published:** 2021-02-05

**Authors:** Ching-Hsuan Liu, Cheng-Hua Lu, Shu Hui Wong, Liang-Tzung Lin

**Affiliations:** ^1^ Graduate Institute of Medical Sciences, College of Medicine, Taipei Medical University, Taipei, Taiwan; ^2^ Department of Microbiology & Immunology, Dalhousie University, Halifax, NS, Canada; ^3^ International Ph.D. Program in Medicine, College of Medicine, Taipei Medical University, Taipei, Taiwan; ^4^ Department of Microbiology and Immunology, School of Medicine, College of Medicine, Taipei Medical University, Taipei, Taiwan

**Keywords:** Severe Acute Respiratory Syndrome Coronavirus 2 (SARS-CoV-2), Coronavirus Disease 2019 (COVID-19), treatment, therapeutic, antiviral

## Abstract

By December 2020, the COVID-19 pandemic had caused more than 74 million confirmed cases and 1.6 million related deaths around the world. However, only a few drugs have been approved in certain areas and for use in conditional patients, and the vaccine candidates were only recently approved or authorized for emergency use without being fully implemented worldwide, suggesting that we are yet to reach effective control of the current outbreak as its uninhibited transmission continues precariously. Over the past few months, several therapeutic candidates have been proven ineffective in large clinical trials, while some other agents exhibited promising preliminary results. Meanwhile, the investigation of SARS-CoV-2-specific antivirals is underway. Despite still being preclinical, these agents could be beneficial for the long-term control of COVID-19 and deserve more research focus. In this article, we update the current status of therapeutic candidates that have been examined for COVID-19 management, including the virus-targeting inhibitors and host-targeting agents, with their antiviral efficacy *in vitro*, *in vivo*, and in clinical studies. Finally, we highlight the current challenges and future prospect of developing potent therapeutic agents against COVID-19.

## Introduction

Coronavirus Disease 2019 (COVID-19), an infectious disease resulting from the Severe Acute Respiratory Syndrome Coronavirus 2 (SARS-CoV-2) infection, has ignited the current global pandemic since December of 2019 when it first emerged in Wuhan, China. Although there were several outbreaks of coronavirus throughout history, including the Severe Acute Respiratory Syndrome (SARS) during 2002-2003 in South East Asia and Middle East Respiratory Syndrome (MERS) in 2012 in Middle East and 2015 in Korea (1), none of them caused such a huge widespread burden to public health as the current one does. By December 2020, there were over 74 million confirmed cases and 1.6 million deaths globally (2), but only a few antivirals have been approved or authorized for emergency use to treat COVID-19 patients.

Coronaviruses are a group of enveloped, positive-sense, single-stranded RNA viruses that infect a variety of mammal and avian animals including civets, bats, humans, chickens, etc. Unlike HCoV-229E, NL63, OC43, and HKU-1, which typically cause common cold with mild upper respiratory symptoms in humans, SARS-CoV, MERS-CoV and the latest SARS-CoV-2 are associated with more complicated and severe clinical signs and symptoms. In acute SARS-CoV-2 infection, individuals usually show mild symptoms such as cough and fever. After the initial 2-14 days of incubation period, most COVID-19 patients develop pneumonia with dyspnea and hypoxemia, which can progress into acute respiratory disease (3). The fatality rate varies in different regions, ranging from 0.9–9.1% in the most affected countries (4). SARS-CoV-2 is mainly transmitted through respiratory droplets and close contact from person to person (3). Asymptomatic (5) or presymptomatic (6) carriers can also spread the virus, causing a big challenge to the control of COVID-19. Consequently, antivirals and vaccine development become the major task for further control of the current outbreak.

## Virology

**Figure 1:**
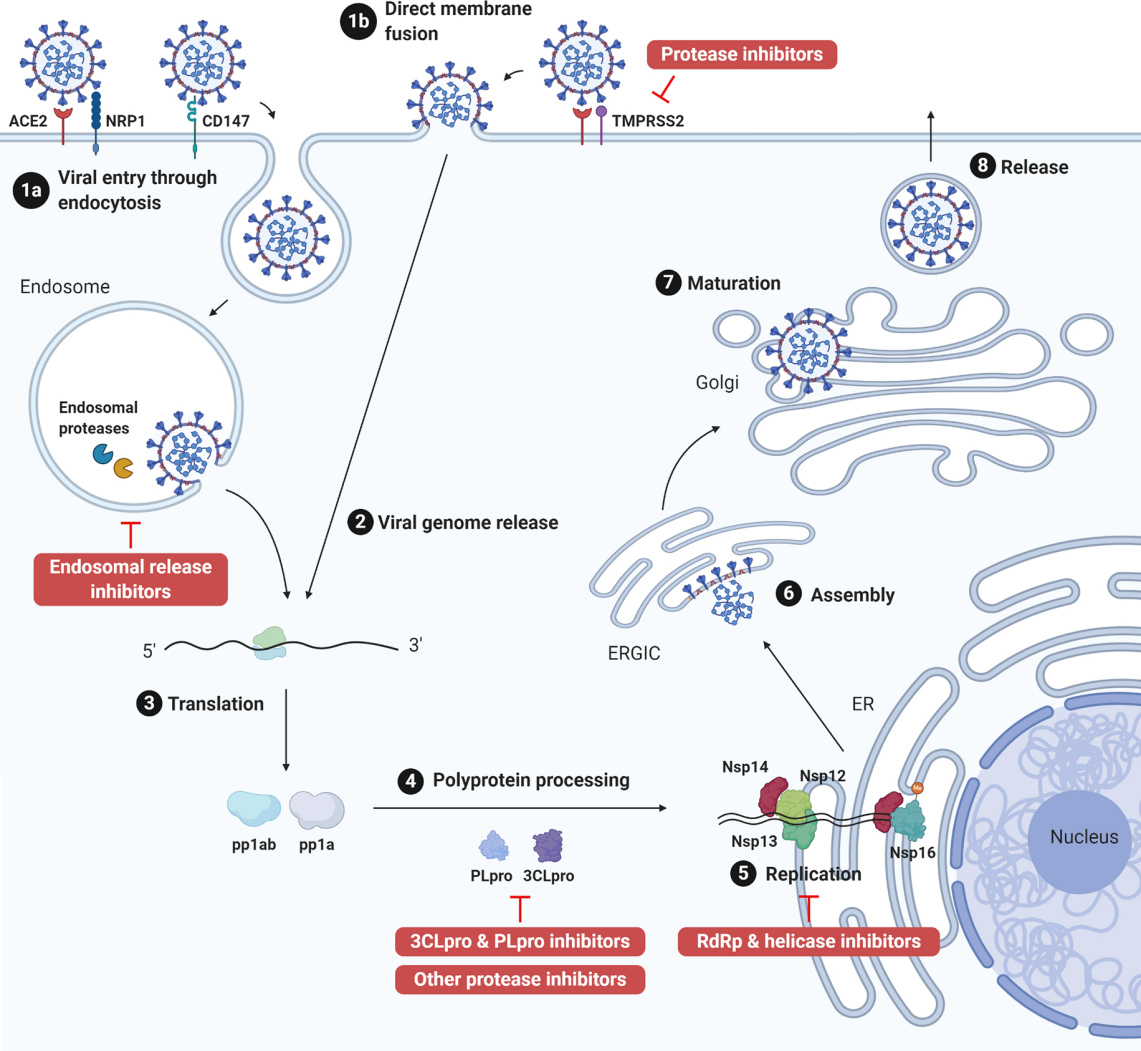
SARS-CoV-2 life cycle and antiviral targets. SARS-CoV-2 infection initiates from viral binding to the host cell receptors angiotensin conversion enzyme 2 (ACE2) or CD147. The virus enters the cell either through endocytosis (1a), after which the virus is processed by endosomal proteases and fuses with endosomal membrane, or direct fusion with the plasma membrane in the presence of transmembrane protease serine 2 (TMPRSS2) (1b). Viral genome is released into the cytoplasm (2) and translated to polyprotein 1ab (pp1ab) and polyprotein 1a (pp1a) (3). Pp1a and pp1ab are further cleaved into 16 nonstructural proteins (nsp1-16) by the viral papain like protease (PLpro, nsp3) and 3C-like protease (3CLpro, nsp5) (4). Viral replication is initiated by replication complex (Nsp12-14) and RNA methyltransferase (nsp14, nsp16) in the endoplasmic reticulum (ER) (5). After which, new viral particles are assembled in the ER-Golgi intermediate compartment (ERGIC) (6) followed by spike protein glycosylation and maturation in the Golgi apparatus (7). Finally, progeny virions are released from the host cell through exocytosis (8). Created with Biorender.com.

The genome of coronaviruses ranges from 27 to 32 Kb and follows an invariant 5’-replicase-S-E-M-N-3’ organization containing a large replicase gene and four structural genes, nucleocapsid (N), glycoprotein spike (S), membrane protein (M), and envelope protein (E). Ribosomal frameshifting-dependent translation of the replicase gene ORF1a and ORF1b forms two coterminal polyproteins pp1a and pp1ab, which undergo autoproteolytic cleavage to produce 16 non-structural proteins (nsp1-16), including viral proteases, RNA dependent RNA polymerase (RdRp), and other viral accessory proteins (1). It is evident that both SARS-CoV and SARS-CoV-2 utilize the human angiotensin-converting enzyme 2 (ACE2) type I membrane protein as a receptor for viral entry (7). Coronaviruses enter either through direct membrane fusion with the presence of Transmembrane Serine Protease 2 (TMPRSS2) on the cell surface or through clathrin-mediated endocytosis, which requires endosomal proteases to prime the viral particle for viral-endosomal membrane fusion (8). Recent studies also suggested that CD147 could serve as an alternative receptor in lung, kidney, and ACE2-deficient cells such as CD4+ and CD8+ T cells, and allow SARS-CoV-2 entry through the endocytosis route (9). In addition, neuropilin-1 (NRP1) has been reported as an entry factor that binds to the furin-cleaved S1 fragment and enhances SARS-CoV-2 infection in the respiratory and olfactory epithelium (10, 11). After entry, viral genome is released into the cytoplasm and translated to primary viral polyproteins pp1a and pp1ab, which self-process *via* the nsp3 papain-like protease (PLpro) and nsp5 3C-like protease (3CLpro), or the so-called main protease (Mpro), into various mature viral proteins that form the replication complex and membrane-associated complex (8). The replication complex comprising viral RdRp (nsp12), helicase (nsp13), exoribonuclease (nsp14), and RNA methyltransferases (nsp14 and 16) then initiates viral replication and transcription, producing new full-length nucleocapsid-encapsidated viral RNA genome in the endoplasmic reticulum (ER) (8). The nucleocapsid core becomes enveloped through the ER-Golgi intermediate compartment, and the viral S protein is lastly glycosylated and cleaved in the Golgi apparatus before mature progeny virions are released through exocytosis for the next round of viral life cycle (8) (**Figure 1**).

## Antiviral Development

Given the need to develop antivirals against coronaviruses, numerous drug candidates are being evaluated for their therapeutic effect in COVID-19. These include viral polymerase inhibitors, protease inhibitors, helicase inhibitors, and host targeting agents. This review focuses on drugs that either have predicted/*in vitro*/*in vivo* antiviral activities against SARS-CoV-2 or related coronaviruses, or are being investigated in COVID-19 clinical setting (**Table 1**). Of note, several drugs have been proven non-effective in randomized controlled trials, including the human immunodeficiency virus (HIV) protease inhibitors lopinavir/ritonavir (LPV/r) (50) and darunavir/cobicistat (DRV/c) (31), and the anti-malarial agents chloroquine (CQ) and hydroxychloroquine (HCQ) (50, 41). Although many of these have been discontinued as mono-therapeutic agents for COVID-19 treatment, agent such as LPV/r is still being assessed in combination with other drugs.

**Table 1:** Therapeutic candidates for COVID-19.

Class	Drug	Antiviral effect		
		*In vitro*	*In vivo*	RCT
Polymerase inhibitor	Ribavirin	EC_50_ = 109.50 μM, CC_50_ > 400 μM, SI > 3.65; MOI = 0.05, 48 hpi, Vero E6 (12)	N/A	IFN-β1b (subcutaneous), LPV/r, ribavirin: faster viral clearance and reduced inflammatory response (13)
	Favipiravir	EC_50_ = 61.88 μM, CC_50_ > 400 μM, SI > 6.46; MOI = 0.05, 48 hpi, Vero E6 (12)	Modest reduction of viral load in hamsters (14)	Higher recovery rate and faster viral clearance in moderate COVID-19 (15, 16); cannot rescue severe COVID-19 (16) Approval in China, Russia, and India (17)
	Remdesivir	EC_50_ = 0.77 μM, CC_50_ > 100 μM, SI > 129.87; MOI = 0.05, 48 hpi, Vero E6 (12)	Improved clinical outcome in rhesus macaques (20)	Shorter time to recovery (18); few clinical benefits in severe COVID-19 (19) US FDA approval in hospitalized patients (21)
	Sofosbuvir & HCV NS5A inhibitors	Sofosbuvir: EC_50_ = 6.2 μM, CC_50_ = 381 μM, SI = 61; MOI = 0.1, 48 hpi, Huh-7 (23) EC_50_ = 9.5 μM, CC_50_ = 512 μM, SI = 54; MOI = 0.1, 48 hpi, Calu-3 (23) Daclatasvir: EC_50_ = 0.8 μM, CC_50_ = 31 μM, SI = 39; MOI = 0.01, 24 hpi, Vero (23) EC_50_ = 0.6 μM, CC_50_ = 6.1 μM, SI = 47; MOI = 0.1, 48 hpi, Huh-7 (23) EC_50_ = 1.1 μM, CC_50_ = 3.0 μM, SI = 34; MOI = 0.1, 48 hpi, Calu-3 (23)	N/A	Sofosbuvir/daclatasvir: faster recovery and lower mortality (22) Sofosbuvir/velpatasvir: in progress
Protease inhibitor	Boceprevir	EC_50_ = 1.31 μM, CC_50_ >100 μM, SI > 76.3; 60 TCID_50_, 5 dpi, Vero 76 (24)	N/A	N/A
	α-ketoamide (compound 13b)*	EC_50_ = 4~5 μM, CC_50_ unknown; MOI = 0.05, Calu-3 (25)	N/A	N/A
	Peptidomimetic aldehydes 11a & 11b*	11a: EC_50_ = 0.53 μM, CC_50_ > 100 μM, SI > 189; MOI = 0.05, 24 hpi, Vero E6 (26) 11b: EC_50_ = 0.72 μM, CC_50_ > 100 μM, SI > 139; MOI = 0.05, 24 hpi, Vero E6 (26)	N/A	N/A
	GC-376*	EC_50_ = 3.37 μM, CC_50_ >100 μM, SI > 29.7; 60 TCID_50_, 5 dpi, Vero 76 (24)	N/A	N/A
	Calpain inhibitors*	Calpain inhibitor II: EC_50_ = 2.07 μM, CC_50_ >100 μM, SI > 48.3; 60 TCID_50_, 5 dpi, Vero 76 (24) Calpain inhibitor XII: EC_50_ = 0.49 μM, CC_50_ >100 μM, SI > 204; 60 TCID_50_, 5 dpi, Vero 76 (24)	N/A	N/A
	Lopinavir/ Ritonavir (LPV/r)	EC_50_ = 26 μM, CC_50_ = 49.75 μM, SI = 1.9; MOI = 0.02, 48 hpi, Vero E6 (29)	Lower clinical scores but no effect on viral titers in ferrets (30)	No clinical benefits (27, 28)
	Darunavir/ Cobicistat (DRV/c)	EC_50_ >100 μM (inactive), CC_50_ >100 μM; MOI = 0.01, 48 hpi, Caco-2 (32)	N/A	No impact on viral clearance (31)
Host-targeting agent	Interferons (IFNs)	IFN-α A/D (pretreatment): EC_50_ = 1.35 IU/ml; MOI = 0.01, 22 hpi, Vero (33) IFN-β1a (pretreatment): EC_50_ = 0.76 IU/ml; MOI = 0.01, 22 hpi, Vero (33) IFN-β1a (posttreatment): EC_50_ = 1.971 IU/ml; MOI = 0.001, 48 hpi, Vero E6 (35) IFN-β1 (2,000 IU/ml) & IFN-λ (300 ng/ml): Prevented infection in T84 & Caco-2 (37)	N/A	IFN-α2b (aerosol): Faster viral clearance and reduced inflammatory response (34) IFN-β1b (subcutaneous), LPV/r, ribavirin: Faster viral clearance and reduced inflammatory response (13) IFN-β1a (subcutaneous): Increased discharge rate and decreased mortality in severe COVID-19 (36) IFN-κ (aerosol) plus TFF2: Faster recovery, viral clearance, and discharge (38) IFN-λ: In progress
	Serine protease inhibitors	Camostat: EC_50_ = 87 nM, **entry** in Calu-3 (39) Nafamostat: EC_50_ = 5 nM, **entry** in Calu-3 (39)	N/A	In progress
	Dexamethasone	N/A	N/A	Lower mortality in COVID-19 patients who required respiratory support (40)
	Losartan	N/A	N/A	In progress
	Chloroquine (CQ)/Hydroxychloroquine (HCQ)	CQ: Inactive in Calu-3 cells (43) EC_50_ = 2.71/3.81/7.14/7.36 μM, CC_50_ = 273.20 μM, SI = 100.81/71.71/38.26/37.12; MOI = 0.01/0.02/0.2/0.8, 48 hpi, Vero E6 (44) EC_50_ = 1.13 μM, CC_50_ > 100 μM, SI > 88.50; MOI = 0.05, 48 hpi, Vero E6 (12) EC_50_ = 5.47 μM; MOI = 0.01, 48 hpi, Vero (46) HCQ: EC_50_ = 4.51/4.06/17.31/12.96 μM, CC_50_ = 249.50 μM, SI = 55.32/61.45/14.41/19.25; MOI = 0.01/0.02/0.2/0.8, 48 hpi, Vero E6 (44) EC_50_ = 0.72 μM; MOI = 0.01, 48 hpi, Vero (46)	CQ: N/A HCQ: Lower clinical scores but no effect on viral titers in ferrets (30) No antiviral activity in hamsters (14, 45) No antiviral activity in macaques (45, 47)	No clinical benefits (41, 42)
	Arbidol	EC_50_ = 4.11 μM, CC_50_ = 31.79 μM, SI = 7.73; MOI = 0.05, 48 hpi, Vero E6 (49)	N/A	No clinical benefits (48)

N/A, not available; RCT, randomized controlled trial.

*SARS-CoV-2 Mpro inhibitors.

### RdRp Inhibitors/Nucleotide Analogs

#### Ribavirin

Ribavirin is an FDA-approved broad-spectrum antiviral prodrug that inhibits viral replication in several proposed mechanisms (51). As a guanosine analog, its metabolite ribavirin monophosphate (R-MP) has been reported to competitively inhibit host cellular inosine monophosphate dehydrogenase (IMPDH), which results in GTP depletion and affects downstream cellular and viral functions. The triphosphate derivative (R-TP) inhibits viral RdRp or creates viral mutagenesis by substituting GTP, although the activities could vary among viruses. *In vitro* studies reported that the anti-SARS-CoV activity of ribavirin is weak in Vero cells (EC_50_ > 1 mg/ml) (52, 53) but appears better in human cell lines (EC_50_ < 10 μg/ml) (54). However, the effect of ribavirin in SARS patients appeared inconclusive and possibly harmful due to its toxicity (55), and later mouse studies demonstrated that ribavirin did not increase the survival rate of infected mice (56, 57). Similarly, ribavirin could not inhibit MERS-CoV replication *in vitro* (58). As for SARS-CoV-2, high concentration of ribavirin was required to suppress the infection (EC_50_ = 109.50 μM, SI > 3.65) (12). These findings suggest that ribavirin as a monotherapy is insufficient to inhibit coronaviruses and that combinatorial therapies are required, such as with interferon (IFN)-α for hepatitis C virus (HCV) (59), with LPV/r viral protease inhibitors for SARS-CoV (60), and with LPV/r and IFN-α for MERS-CoV (61). A trial evaluating the combination of ribavirin, LPV/r, and IFN-β1b (NCT04276688) is described below.

#### Favipiravir

Favipiravir is a pyrazine-derived prodrug that is phosphoribosylated to its active form favipiravir-ribofuranosyl-5’-triphosphate (F-RTP), which incorporates into nascent viral RNA through competition with purine nucleotides and inhibits viral replication (62, 63). As a licensed antiviral for influenza in Japan and China, past studies have shown that favipiravir provides a broad-spectrum antiviral activity against multiple strains of influenza virus types A, B, and C (63–67) and a wide range of RNA viruses [reviewed in (68)] *in vitro* and *in vivo*.

Like ribavirin, high dose of favipiravir (EC_50_ = 61.88 μM, SI > 6.46) was required to inhibit SARS-CoV-2 infection *in vitro* (12), and a recent preprint suggested that the drug only decreased 0.9 log_10_ viral RNA copies/mg lung tissue in infected hamsters without affecting the pathology or preventing transmission (14). Nonetheless, favipiravir has been investigated in several clinical trials with preliminary results. In a non-randomized open-label before-after controlled trial (ChiCTR2000029600), patients who received favipiravir plus IFN-α had a higher improvement rate in their chest imaging and faster viral clearance compared to the control group receiving LPV/r plus IFN-α (15). A randomized open-label controlled trial (ChiCTR2000030254) compared favipiravir treatment with arbidol, an indole-derivative small molecule anti-influenza virus drug, and reported in a preprint that moderate COVID-19 patients treated with favipiravir had higher recovery rate and required less auxiliary (16). However, neither of the two drugs effectively rescued severe disease patients, indicating the restricted use of favipiravir in moderate COVID-19 patients to prevent deterioration (16). Based on the preliminary results, favipiravir has been approved for the treatment of COVID-19 in China, Russia, and India (17).

#### Remdesivir

Remdesivir (GS-5734) is a broad-spectrum adenosine analog prodrug that inhibits early viral RNA synthesis by causing delayed chain termination (69–72). The compound exhibits antiviral activities against Ebola virus (69, 71, 73), respiratory syncytial virus (RSV) (69, 73), Nipah virus, parainfluenza virus (73), and a panel of coronaviruses including endemic human-CoVs, SARS-CoV, MERS-CoV, bat-CoVs, and murine hepatitis virus (MHV) (74–76). The drug has been evaluated in clinical trials with Ebola virus disease patients, but it appeared less effective than Ebola virus-specific monoclonal antibodies (77).

In coronavirus models, it has been demonstrated that prophylactic or early therapeutic administration of remdesivir is critical for inhibiting viral replication. Remdesivir given 1 day pre-infection and 1 day post-infection (dpi) in SARS-CoV-infected mice both reduced the lung viral titer and SARS-CoV-induced lung pathology, while treatment given 2 dpi only reduced viral load without improving disease outcome (74). Remdesivir treatments in MERS-CoV-infected mice (78) and rhesus macaques (79) also demonstrated that both prophylactic and early therapeutic treatments decreased the viral load, clinical signs, and pathology, with the prophylactic approach being more protective.

As for SARS-CoV-2, *in vitro* data indicate high antiviral potency of remdesivir (EC_50_ = 0.77 μM, SI > 129.87) (12), and the drug improved clinical outcome of SARS-CoV-2-infected rhesus macaques when given at 12 hours post-infection (hpi) (20). The drug has received Emergency Use Authorization (EUA) in the United States (US) as well as statutory approval in Japan for the treatment of COVID-19, based on results from phase 3 trials supported by the US National Institute of Allergy and Infectious Diseases (NIAID) (NCT04280705; Adaptive COVID-19 Treatment Trial, ACTT 1) and Gilead (NCT04292899; SIMPLE trial) (80, 81). ACTT 1 is a multi-center, double-blind, randomized, placebo-controlled trial which suggested that 10-day remdesivir shortens the time to recovery but has no significant impact on mortality based on its preliminary results (18), whereas SIMPLE trial compared a 5-day course and a 10-day course of remdesivir and reported that the clinical outcomes were similar in severe COVID-19 patients (82). However, a recently published multi-center, randomized, placebo-controlled trial found that remdesivir did not lower the viral load and did not provide clinical benefits in patients with severe COVID-19 (19). Nonetheless, the US FDA has approved the use of remdesivir in hospitalized patients in October 2020, but the rest of the population is still covered under the EUA issued in May 2020 (20).

On the other hand, Yan and Muller recently suggested that the parental nucleoside of remdesivir, GS-441524, may be superior to remdesivir for the treatment of COVID-19 based on their pharmacokinetic profiles (83). Bioactivation of remdesivir requires enzymes that are predominantly expressed in the liver rather than the lungs and could possibly explain the liver-related adverse effects in remdesivir-treated COVID-19 patients. Additionally, esterases and phosphatases in the serum facilitates premature hydrolysis of the McGuigan prodrug on remdesivir, resulting in the presence of GS-441524 as the predominant species in serum after remdesivir administration (69, 20). Therefore, further investigation of GS-441524 for the treatment of COVID-19 could be considered to prevent deferential bioactivation and off-target effect of the prodrug (83).

#### Sofosbuvir and HCV NS5A Inhibitors

Sofosbuvir is a licensed uridine nucleotide analog prodrug that competitively blocks HCV NS5B polymerase and causes RNA chain termination (84). Since SARS-CoV-2 and HCV are both positive-sense RNA viruses, the use of HCV polymerase inhibitors is expected to be effective for SARS-CoV-2 to some extent. Clinically used with sofosbuvir for the treatment of hepatitis C (85), daclatasvir is one of the HCV NS5A inhibitors that interferes with HCV replication complex (86).

*In silico* docking analyses reported that sofosbuvir bound to SARS-CoV (87) and SARS-CoV-2 (88) RdRp active sites, suggesting potential antiviral activities. *In vitro* data displayed on preprint server demonstrated that sofosbuvir did not inhibit SARS-CoV-2 in Vero cells, but was active in human hepatoma Huh-7 cells (EC_50_ = 6.2 μM, SI = 61) and human lung adenocarcinoma Calu-3 cells (EC_50_ = 9.5 μM, SI = 54) (23). Meanwhile, daclatasvir inhibited SARS-CoV-2 in all three cell lines (EC_50_ = 0.6~1.1 μM, SI = 34~47) (23). Several trials are ongoing to evaluate sofosbuvir/daclatasvir in COVID-19 patients. A small multi-center, double-blind, randomized, controlled trial (IRCT20200128046294N2) was recently completed and reported a faster recovery in moderate to severe COVID-19 patients who received sofosbuvir/daclatasvir plus LPV/r, compared to those who received only LPV/r (22). Furthermore, meta-analysis of the combined results from this study and the other ones in Iran favored the use of sofosbuvir/daclatasvir with significantly reduced time to recovery and mortality (22). A larger multi-center, double-blind, randomized, controlled trial (IRCT20200624047908N1) is underway to validate the results. In addition, the combination of sofosbuvir/velpatasvir (another HCV NS5A inhibitor) will be evaluated in another single-center, single-blind, randomized, controlled trial (IRCT20130812014333N145) (89).

### Protease Inhibitors

#### HCV NS3/4A Protease Inhibitors

Based on several preliminary structural analyses in preprints (90, 91), HCV NS3/4A shares a three-dimensional similarity with the SARS-CoV-2 Mpro, suggesting a potential of investigating HCV protease inhibitors in SARS-CoV-2 infection. Several licensed (simeprevir, paritaprevir, grazoprevir, glecaprevir, boceprevir, telaprevir) and investigational (sovaprevir, vaniprevir, danoprevir) HCV protease inhibitors were predicted to bind to the SARS-CoV-2 Mpro active site (90, 91). Enzymatic and binding assays further revealed that boceprevir (IC_50_ = 4.13 µM) and narlaprevir (another licensed HCV protease inhibitor; IC_50_ = 4.73 µM) inhibited Mpro more potently than simeprevir (IC_50_ = 13.74 µM), and the antiviral activity of boceprevir against SARS-CoV-2 (EC_50_ = 1.31 µM, SI > 76.3) was confirmed *in vitro* (24). Currently, there are no large randomized trials evaluating FDA-approved HCV protease inhibitors in COVID-19 patients. Nonetheless, agents such as boceprevir which is already licensed and displayed anti-SARS-CoV-2 *in vitro* may be suitable candidates for clinical or at least *in vivo* studies.

#### CoV Mpro and PLpro Inhibitors

Protease inhibitors designed for coronaviruses are also being investigated. These drug candidates could be more specific but are mostly preclinical. Some examples with anti-SARS-CoV-2 activity shown *in vitro* are described here. α-ketoamides are a class of peptidomimetic compounds that were synthesized to inhibit coronavirus Mpro and enterovirus 3C protease, exhibiting antiviral effects against SARS-CoV (EC_50_ = 5.8 μM), MERS-CoV (EC_50_ = 0.0047 μM), HCoV-229E (EC_50_ = 11.8 μM), Enterovirus 71 (EC_50_ = 9.8 μM) (92), and the current SARS-CoV-2 (compound 13b, EC_50_ = 4~5 μM) (25). Peptidomimetic aldehydes 11a and 11b were also designed and synthesized based on the structure of SARS-CoV-2 Mpro, and both compounds potently inhibited SARS-CoV-2 (11a: EC_50_ = 0.53 μM, SI > 189; 11b: EC_50_ = 0.72 μM, SI > 139), with compound 11a displaying better pharmacokinetic profile (26). The bisulfite adduct GC-376, an investigational veterinary drug that inhibits feline infectious peritonitis virus Mpro and a number of other viruses (93–95), also inhibits SARS-CoV-2 Mpro (IC_50_ = 0.03 µM) and effectively precludes SARS-CoV-2 infection (EC_50_ = 3.37 μM, SI > 29.7) (24). The same study also revealed two other SARS-CoV-2 Mpro inhibitory compounds, calpain inhibitor II (IC_50_ = 0.97 µM) and XII (IC_50_ =0.45 µM), that exhibit anti-SARS-CoV-2 activity (EC_50_ = 2.07 µM, SI > 48.3 and EC_50_ = 0.49 µM, SI > 204, respectively) (24). The high potency and specificity of these compounds imply their potential to be further investigated and developed as clinical drugs. In addition, PLpro inhibitors that have exhibited antiviral activities against other CoVs may be worth investigating due to the conserved structures of CoV PLpro (96).

#### Helicase Inhibitors

Due to the predicted similarity and conserved active sites in the nsp13 helicase of SARS-CoV and SARS-CoV-2, helicase inhibitors previously shown to inhibit SARS-CoV could be of potential value (97). For example, the adamantane-derived Bananin was shown to inhibit SARS-CoV ATPase (IC_50_ = 2.3 µM) and helicase (IC_50_ = 3.0 µM) activities and viral replication (EC_50_ < 10 μM, CC_50_ > 300 μM) (98). Another compound, SSYA10-001, was also shown to inhibit SARS-CoV helicase (IC_50_ = 5 µM) and replication (EC_50_ = 8.95 μM, CC_50_ > 250 μM) (99). SSYA10-001 also inhibits the replication of MERS-CoV (EC_50_ ~ 25 μM) and MHV (EC_50_ ~ 12 μM), which have conserved active sites in their helicases as that of SARS-CoV (100). These compounds, however, have not been examined in SARS-CoV-2 infection models and merits further investigation.

#### Lopinavir/Ritonavir

LPV/r is a combination of two protease inhibitors used for the treatment of HIV infection (101–103). Lopinavir is an uncleavable peptidomimetic of the linkage peptide in HIV gag-pol polyprotein that binds to HIV protease and inhibits its activity. Ritonavir, also a HIV protease inhibitor, mainly functions as an inhibitor of cytochrome P450 CYP3A4 isoenzyme in LPV/r. Low-dose ritonavir in the coformulation improves the pharmacokinetic profile of lopinavir through inhibition of its CYP3A-mediated metabolism (100–103).

Previously investigated during the SARS outbreak, LPV/r demonstrated synergistic effect in combination with ribavirin, and addition of LPV/r to ribavirin and corticosteroid was shown to improve the clinical outcome compared to the control group without LPV/r treatment (60). Another retrospective cohort study also indicates that the addition of LPV/r to ribavirin and corticosteroid reduced the mortality rate and intubation rate compared to a matched cohort receiving only ribavirin and corticosteroid (104). *In silico* analysis suggested that LPV/r interacts with SARS-CoV 3CL protease and binds to its active site, forming a flap closing conformation that has been observed in HIV-1 protease when an inhibitor is bound (105).

As for its effect on MERS-CoV, LPV/r plus IFN-β combination could not prevent mice from MERS-CoV infection, but as a treatment improved pulmonary function without affecting the viral load or severe lung pathology, indicating a less effective role than remdesivir (78). However, in a marmoset MERS-CoV model, LPV/r alone and LPV/r plus IFN-β were reported to reduce lung viral load and improve clinical outcome in comparison with untreated animals (106). A randomized, placebo-controlled, double-blind trial (NCT02845843; MIRACLE) to investigate the LPV/r and IFN-β1b combination in hospitalized MERS patients was initiated and just recently completed (107), but the results are not yet available.

In the context of SARS-CoV-2 infection, despite limited preclinical data in Vero E6 cells (EC_50_ = 26 μM, SI = 1.9) (29) and in ferrets (30), several clinical trials were underway to evaluate the effect of LPV/r in COVID-19 patients. A single-center, randomized, controlled, open-label trial in China (ChiCTR2000029308) compared the standard of care with LPV/r plus standard of care for 14 days in severe COVID-19 patients but found no significant difference in the median time to clinical improvement, 28-day mortality, and length of ICU stay, despite numerical reductions (27). The study design and the small sample size prompted a discussion that results from this study were statistically underpowered and inconclusive to exclude further investigation of the drug (108–113), suggesting the need to initiate trials with bigger sample sizes and earlier treatment. However, as a participant of the WHO Solidarity Trial, the RECOVERY trial conducted in the United Kingdom (EudraCT 2020-001113-21/NCT04381936) reported that LPV/r monotherapy provided no clinical benefit in hospitalized COVID-19 patients (28). The study compared 1596 patients randomized to LPV/r treatment and 3376 patients who received only standard of care and found no significant difference in the 28-day mortality, the risk of progression to mechanical ventilation, and length of hospital stay (28). Along with other interim trial results, WHO has announced to discontinue the treatment arm of LPV/r monotherapy for COVID-19 patients in the Solidarity Trial (50).

#### Darunavir/Cobicistat

The nonpeptidic drug darunavir (TMC114) is another licensed HIV protease inhibitor. Darunavir has a higher binding affinity and potency over other HIV protease inhibitors (114, 115), and it prevents the viral protease from dimerizing and obtaining its proteolytic activity (116). Darunavir is often prescribed with cobicistat as a CYP3A inhibitor (DRV/c) for treatment of HIV-1 infection (117, 118).

In contrast to LPV/r, DRV/c was never tested for its antiviral activity in SARS-CoV or MERS-CoV infection. As for its effect against SARS-CoV-2 *in vitro*, the drug did not show any inhibition in human colorectal adenocarcinoma Caco-2 cells (EC_50_ >100 μM) (32). Despite the lack of preclinical evidence, a few clinical trials were initiated to evaluate its effect in COVID-19 patients. Of which, results from a single-center, randomized, open-label controlled trial in China (NCT04252274) concluded that 5-day DRV/c treatment had no impact on viral clearance compared to the control group in mild COVID-19 patients (31), indicating its ineffectiveness for the disease.

### Host-Targeting Agents

#### Interferons

The antiviral activity of IFNs has been well studied in coronaviruses. For SARS-CoV, IFN-β1b showed the highest *in vitro* antiviral activity (EC_50_ = 9.2 and 21.0 IU/ml, SI > 1,087 and 476 against the Hong Kong and FFM-1 isolates, respectively) compared to IFN-α2b (EC_50_ = 880 and 1,530 IU/ml, SI > 11.4 and 6.5) and IFN-γ1b (EC_50_ > 10,000 IU/ml for both isolates) in Caco-2 cells (119). *In vivo*, IFN-α B/D treatment starting 4 hpi for 3 days successfully reduced viral titer in BALB/c mice (120). In another study, a single dose of IFN-β given at 6 hpi protected the mice from lethal SARS-CoV challenge, preventing the delayed type I IFN signaling that contributes to SARS immunopathology (121). In an uncontrolled small clinical study, patients who received IFN alfacon-1 for 8-13 days in addition to corticosteroid treatment exhibited better clinical outcome than those who were treated with corticosteroid alone (122).

Similarly for MERS-CoV, IFN-β also displayed the highest potency amongst other IFNs against MERS-CoV in Vero cells (58, 123). On the other hand, IFN-λ was shown to inhibit MERS-CoV replication in human respiratory epithelium (124). In animal studies, marmosets treated with IFN-β1b 8 h after viral challenge exhibited better clinical outcome (106). IFN-α2b and ribavirin treatment started 8 hpi also improved the clinical outcome in rhesus macaques with MERS-CoV challenge (125). In one clinical trial in MERS patients, IFN treatment (IFN-α2a, IFN-α2b, or IFN-β1a) alone or with ribavirin did not improve the survival rate or viral clearance (126); however, 60% of the IFN-treated patients also received corticosteroid, which might have suppressed IFN signaling (127). The therapeutic effect of IFN-β1b plus LPV/r is yet to be determined from the results of the MIRACLE trial (107).

As for SARS-CoV-2, IFN-α A/D (EC_50_ = 1.35 IU/ml) and IFN-β1a (EC_50_ = 0.76 IU/ml) pretreatment inhibited viral replication *in vitro* at low EC_50_ values (33). When administered at 1 hpi, IFN-β1a also inhibited viral infection in Vero E6 cells (EC_50_ = 1.971 IU/ml) (35), although the MOI used was relatively low. In human colorectal adenocarcinoma T84 and Caco-2 cells and human colon organoids, both type I (IFN-β1) and type III (IFN-λ) IFNs prevented SARS-CoV-2 infection (37). Interestingly, SARS-CoV-2 infection significantly upregulated the production of IFN-λ but not IFN-β1 in colon organoids, suggesting a critical role of type III IFN response in controlling the infection in human intestinal cells (37).

In recent clinical studies, IFN monotherapy and combination therapies were investigated. A study in the Union Hospital in Wuhan suggested that IFN-α2b aerosol with or without arbidol reduced the duration of viral clearance and inflammatory response compared to arbidol monotherapy (34). A triple combination of IFN-β1b (subcutaneous injection), LPV/r, and ribavirin also led to similar results compared to LPV/r monotherapy in a multi-center, open-label, randomized, controlled trial (NCT04276688) (13). Another study reported that the addition of IFN-β1a to standard of care did not improve the overall clinical outcome but increased the discharge rate on day 14 and decreased the 28-day mortality in patients with severe COVID-19 (36). More trials of type I IFNs in combination with LPV/r (e.g., WHO Solidarity DisCoVeRy trial) or remdesivir (e.g., NIAID ACTT 3), as well as trials of IFN-λ (NCT04354259)are ongoing. In addition, a pilot study reported that inhalation of IFN-κ plus trefoil factor 2 (TFF2) shortened the time of symptom relief, viral clearance, and hospitalization (38). These findings suggest that IFNs are a promising candidate for COVID-19 treatment. Notably, the route of IFN administration will be a critical issue to consider to achieve the best bioavailability in the target organs (127).

#### Serine Protease Inhibitors

Due to the essential role of serine protease TMPRSS2 in activating SARS-CoV-2 spike for its entry on the cell membrane (7), serine protease inhibitors are also an attractive group of drugs. Camostat, a serine protease inhibitor approved for the treatment of chronic pancreatitis in Japan (128), has been shown to inhibit SARS-CoV entry (129) and partially protected mice from lethal challenge (130). It was recently reported that camostat (EC_50_ = 87 nM) and another serine protease inhibitor nafamostat (EC_50_ = 5 nM) used as an anticoagulant were able to inhibit SARS-CoV-2 entry at low concentrations (39). Several clinical trials investigating these two drugs are underway.

#### Dexamethasone

Dexamethasone is a licensed corticosteroid commonly used for its anti-inflammatory effects (131). The use of corticosteroids in viral pneumonia and acute respiratory distress syndrome has been controversial (132, 133). Although theoretically corticosteroids could alleviate the inflammation of viral pneumonia, several previous studies demonstrated that corticosteroid treatment could delay viral clearance and induce multiple complications, providing no clinical benefits (134). However, preliminary results from the RECOVERY trial suggested that the use of dexamethasone lowered the 28-day mortality in hospitalized COVID-19 patients who required respiratory support (40). Noteworthy, no benefits were observed in patients who did not need oxygen support upon admission (40). Based on this preliminary results, dexamethasone is now recommended for hospitalized COVID-19 patients who are mechanically ventilated or require oxygen supplement (135).

#### Losartan

Losartan is an angiotensin II receptor blocker (ARB) for the treatment of hypertension and diabetic nephropathy. It has been shown that losartan increases the expression level of ACE2 (136, 137), which has a protective role in severe acute lung injury (138). A previous study found that SARS-CoV infection and the viral spike protein downregulate ACE2 expression in the lungs, causing severe lung injury in infected mice (139). The administration of losartan reduced the acute severe lung injury and pulmonary edema in SARS-CoV spike-treated mice (139). Due to the similar receptor usage and pathogenesis of SARS-CoV-2 and SARS-CoV, losartan has been proposed as a tentative treatment for COVID-19 (140, 141). However, increasing ACE2 expression also raises the concern of enhancing SARS-CoV-2 infection, thus careful monitoring and safety evaluation are mandatory. Clinical data of losartan’s therapeutic effect in COVID-19 patients are not yet available. In addition, its use in infected patients with cardiovascular diseases should be continued due to a current lack of evidence for its discontinuation (142).

#### Chloroquine and Hydroxychloroquine

CQ and its derivative HCQ are antimalarial drugs that have also exhibited broad-spectrum antiviral activities by interfering with the endosome-mediated viral entry and the late stages of viral replication, in steps that require an acidic environment (143). CQ and HCQ were reported to possess anti-SARS-CoV activities *in vitro* (120, 144) but appeared ineffective in mouse model (104). The drugs also demonstrated antiviral effects against SARS-CoV-2 in Vero and Vero E6 cells (12, 44, 46) but not in Calu-3 cells (43), and their benefits *in vivo* were highly controversial. HCQ lowered the clinical scores in ferrets but did not affect the viral titers (30), and no antiviral activity was observed in hamsters and macaques (14, 45, 47). Based on the antiviral activities shown *in vitro* and the earliest literature including a brief clinical report in China (145) and the manuscript from Gautret et al., which was later found to have serious methodological issues (146), CQ and HCQ received an EUA in March 2020. However, the positive results were not always replicable in subsequent clinical trials, and the higher quality data from randomized controlled trials suggested that the drugs did not provide any clinical benefits (41, 42). This led to the revocation of EUA (147) and discontinuation of clinical trials of the CQ/HCQ arm (50).

#### Arbidol

Arbidol is a small molecule indole-derivative approved in Russia and China for prophylaxis and treatment against influenza virus infection. The drug inhibits viral infection *via* its intercalation into membrane lipids, which interferes with membrane fusion involved in multiple steps of viral life cycle (148, 149). Previous *in vitro* studies indicated that arbidol is capable of curbing SARS-CoV replication (150) and was the most efficacious anti-influenza drug against SARS-CoV-2 (EC_50_ = 4.11 μM, SI = 7.73) (49). Results from several retrospective trials suggested that arbidol (151, 152) or the combination of arbidol and LPV/r (153) could be beneficial. However, a randomized controlled trial assessing LPV/r and arbidol monotherapies in mild/moderate COVID-19 patients found that both treatments had little clinical benefits as compared to supportive care, with no significant difference in the viral negative conversion rate and symptom improvement (48). Therefore, the clinical effect of arbidol remains questionable.

## Current Challenges and Future Perspective

In order to effectively control the ongoing SARS-CoV-2 global pandemic, vaccination and the development of therapeutics are both indispensable. While a few vaccine candidates are being rolled out, the supply is still limited, and a tremendous effort and amount of time are required to achieve sufficient immunization coverage. Hence, rapid identification of efficacious antivirals remains a top priority to improve management strategies for newly acquired or currently existing infections and minimize fatalities in COVID-19 patients. This highlights the need to employ high-throughput screening and structure-based analyses to fast track the identification of potential candidates. Of note, it is now evident that drugs with promising antiviral activities *in vitro* do not necessarily exert effectiveness in animal models or in humans. Previous experiences in CQ/HCQ (154) and LPV/r (30) have provided an insight that animal studies may help predict the drugs’ effect in a more realistic scenario, although the outcomes can vary between animals and humans. In terms of interpreting clinical data, the design of trials such as the inclusion criteria, treatment assignment, administration route, co-existing treatments, endpoints, and methods of statistical analysis should all be carefully reviewed.

Besides the efficient use of repurposed drugs, SARS-CoV-2-specific antivirals should also be developed for the long-term benefit. Antivirals such as viral polymerase inhibitors and protease inhibitors should be prioritized due to their direct impact on viral replication. This is supported by previous experience with HCV, for which the development of direct acting antivirals (DAAs) substantially improved the therapeutic efficacy (over 95% sustained virological responses) (155). Likewise, the introduction and advancement of combination antiretroviral therapy also successfully improved the survival of HIV-infected patients (156). Hence, development of specific antivirals with different targets in the SARS-CoV-2 life cycle and the use of a combination therapy could be more potent in reducing viral load and prevent severe disease progression.

## Author Contributions

Conceptualization: C-HLiu, C-HLu, and L-TL. Writing—Original Draft: C-HLiu and C-HLu. Writing—Review and Editing: C-HLiu, SHW, and L-TL. Supervision: L-TL. Funding Acquisition: L-TL. All authors contributed to the article and approved the submitted version.

## Funding

C-HLiu has received PhD fellowship from the Canadian Network on Hepatitis C (CanHepC). CanHepC is funded by a joint initiative of the Canadian Institutes of Health Research (CIHR) (NHC-142832) and the Public Health Agency of Canada (PHAC). L-TL is funded by the Ministry of Science and Technology of Taiwan (MOST107-2320-B-038-034-MY3). The funders had no role in study design, data collection and analysis, decision to publish, or preparation of the manuscript.

## Conflict of Interest

The authors declare that the research was conducted in the absence of any commercial or financial relationships that could be construed as a potential conflict of interest.
